# MGDP: Mastering a Generalized Depth Perception Model for Quadruped Locomotion

**DOI:** 10.1002/advs.202524345

**Published:** 2026-04-27

**Authors:** Yinzhao Dong, Ji Ma, Yidan Lu, Jiahui Zhang, Wanyue Li, Yeke Chen, Teng Zhang, Xuechao Chen, Zhangguo Yu, Peng Lu

**Affiliations:** ^1^ Department of Mechanical Engineering The University of Hong Kong Hong Kong SAR China; ^2^ Department of Orthopaedics and Traumatology The University of Hong Kong Hong Kong SAR China; ^3^ College of Mechatronics Beijing Institute of Technology Beijing China

**Keywords:** deep reinforcement learning, quadrupedal robots, robot learning

## Abstract

Perception‐based Deep Reinforcement Learning (DRL) controllers demonstrate impressive performance on challenging terrains. However, existing controllers still face core limitations, struggling to achieve both terrain generality and platform transferability, and are constrained by high computational overhead and sensitivity to sensor noise. To address these challenges fundamentally, we propose a generalized control framework: Mastering a Generalized Contrastive Depth Model (MGDP). We leverage NVIDIA Warp to enable efficient parallel computation of depth images, thereby mitigating the inherent high computational cost. MGDP extracts low‐dimensional terrain feature representations from multi‐modal inputs (depth images and height maps) and integrates an explicit depth map denoising mechanism. This process not only facilitates effective decoupling of perception from dynamics but also significantly reduces the memory. Furthermore, we design terrain‐adaptive reward functions that modulate penalty strengths according to terrain characteristics, enabling the policy to acquire complex locomotion skills (e.g., climbing, jumping, crawling, squeezing) in a single training stage without relying on distillation. Experimental results demonstrate that MGDP not only endows the policy with superior cross‐terrain generalization capability but also enables fast and efficient fine‐tuning across diverse quadruped robot morphologies via its pre‐trained, dynamics‐decoupled perception model. This vigorously advances the development of unified, efficient, and generalized frameworks for quadrupedal locomotion control.

## Abstract 2

This work presents Mastering a Generalized Contrastive Depth Model (MGDP), a perception‐based Deep Reinforcement Learning (DRL) framework for quadruped locomotion. MGDP learns compact terrain features from depth images and height maps with explicit denoising and terrain‐adaptive rewards, enabling robust locomotion skills and efficient transfer across terrains and different robot morphologies.

## 1 Introduction

Deep Reinforcement Learning (DRL) has demonstrated significant power, enabling quadruped robots to execute specialized skills, like bipedal locomotion [1], fall recovery [2], backflip [3], parkour [4]. However, establishing a generalized locomotion control framework that simultaneously achieves robust terrain generality and platform transferability remains a core challenge in current quadruped robotics research. To achieve this goal, existing policies must overcome several major obstacles. First, developing a single, general controller requires the robot to possess robust perception and a fine‐grained understanding of diverse and complex terrains. Second, perception‐based controllers are inherently vulnerable to sensor noise and incur high computational and memory overhead from processing large‐scale image features, thereby restricting both learning efficiency and real‐time deployment. Most critically, the coupling of perception representations, robot dynamic models, and platform‐specific privileged information (e.g., body linear velocity, foot swing height, and terrain height maps, which are available in simulation but not directly measurable on the real robot due to sensor limitations) prevents policies from achieving fast and efficient transfer to new robot platforms with different sizes, masses, joint configurations, and sensor layouts.

Traditional blind‐based controllers rely on proprioception, offering strong robustness and real‐time performance [5, 6, 7]. However, they lack terrain perception, making them incapable of dealing with extreme terrains such as large height variations or sparse footholds. To compensate for this deficiency, perception‐based control utilizing explicit environmental geometry information has become mainstream. Among these, LiDAR‐based controllers [8, 9] provide highly accurate geometric information, but are often burdened by high equipment costs, massive computational overhead for point cloud processing, and inherent sensitivity to environmental dynamic noise. Conversely, depth‐based controllers [4, 10, 11, 12, 13, 14] enable proactive gait planning and adjustment. However, these approaches suffer from multiple restrictions: they often require complex multi‐stage distillation processes, rely heavily on privileged information, and are prone to failure under depth noise or platform changes. More critically, the processing of depth image features introduces substantial computational overhead during large‐scale parallel training, posing a major bottleneck for efficient learning and real‐time deployment.

Based on these limitations, we propose a novel DRL control framework for quadrupedal locomotion: Mastering a Generalized Contrastive Depth Model (MGDP). This framework is designed to address the core challenges of high computational overhead, sensor noise sensitivity, and deep hardware coupling inherent in existing depth‐perception policies. The core of MGDP lies in leveraging a contrastive learning mechanism to extract highly generalized, low‐dimensional terrain feature representations from multi‐modal inputs. This design facilitates the effective decoupling of terrain perception from robot dynamics, combined with an explicit depth map denoising mechanism. Our controller can learn a single, unified general controller that successfully endows it with both superior cross‐terrain generalization capability and rapid cross‐platform transferability without requiring complex multi‐stage distillation. The key contributions can be listed as follows:

- We propose a generalized DRL locomotion framework that utilizes an optimized parallel computation architecture for depth processing, fundamentally mitigating the bottlenecks of high computational overhead and deep hardware coupling in existing perception policies.
- We introduce a robust, generalized depth perception model that achieves effective decoupling of terrain perception from robot dynamics. This is realized by leveraging contrastive learning on multi‐modal inputs to extract low‐dimensional features, integrated with explicit depth map denoising for enhanced policy robustness.
- We design effective Terrain‐Adaptive Reward Functions that enable the policy to execute a unified set of complex locomotion skills and gait adjustments, including crawling, jumping, climbing, and squeezing. This approach allows the policy to adapt to a wider range of challenging terrains without multi‐stage skill distillation.
- We demonstrate that utilizing the pre‐trained MGDP perception model enables fast and efficient transfer (fine‐tuning) across diverse quadruped platforms, significantly advancing the development of unified and transferable locomotion controllers.

## 2 Related Work

### 2.1 Blind‐Based Learning

Blind locomotion relies solely on proprioceptive feedback to achieve dynamic control without any exteroceptive sensing, offering strong robustness and real‐time performance but limited capability to handle complex terrains. Early work, such as RMA [15], addresses this limitation through a teacher–student distillation scheme, where a privileged teacher guides a proprioception‐only student to improve adaptation on unstructured terrains. Subsequent methods further enhance the blind policy's latent state inference. For example, Ji et al. [16] predict key unobservable information (e.g., body velocity, foot height, and foot contact states) from observation history. Nahrendra et al. [6] employ an autoencoder to improve velocity and context estimation consistency, and Luo et al. [5] explicitly infer robot morphology parameters, enabling more reliable transfer across different quadruped platforms. Collectively, these advances illustrate a progressive shift from basic proprioceptive robustness toward richer latent state inference and cross‐robot generalization within blind locomotion.

Although blind locomotion performs well on many irregular terrains, it has difficulty handling extreme conditions involving large gaps, abrupt height changes, or sparse footholds. These limitations motivate the integration of visual or depth perception to provide anticipatory terrain awareness.

### 2.2 Perception‐Based Learning Locomotion

Unlike blind locomotion methods, perception‐based controllers enable legged robots to explicitly interpret external environmental structures such as gap width, rock distribution, obstacle height, and terrain slope. With access to these geometric cues, the robot can adjust its motion strategy before making contact with the terrain, dynamically adapting step length, swing height, and body posture in advance. In recent years, numerous studies have demonstrated the effectiveness of vision‐guided locomotion in demanding parkour environments.

For example, Zhuang et al. [12] propose a multi‐stage skill‐distillation framework that integrates jumping, stepping, and climbing skills into a unified policy. Subsequently, Chane‐Sane et al. [13] and Cheng et al. [4] adopt teacher–student distillation to compress privileged information or directional guidance points into deployable policies, improving stability and generalization under complex obstacle compositions. Moreover, Hoeller et al. [17] take a different pipeline by hierarchically combining multiple pretrained skills to achieve challenging maneuvers such as obstacle traversal and platform jumping, rather than distilling all abilities into a single policy. Despite their strong performance, these controllers share several fundamental limitations. They typically rely on extensive privileged information and involve complex multi‐stage distillation procedures, which substantially increase training cost and memory consumption, while also introducing the risk of performance degradation during knowledge transfer.

To reduce reliance on such distillation pipelines, Luo et al. [11] adopt a transformer architecture combined with NVIDIA Warp [18] training to enable more efficient single‐stage learning. However, its capability remains largely focused on jumping behaviors and does not fully address more diverse parkour skills, such as climbing or crawling. Meanwhile, Lai et al. [19] propose a Perception World Model that does not feed visual features directly into the policy. Instead, it trains a depth‐image–based world model to reconstruct and predict observations. However, the framework does not explicitly handle noise or failure modes in the depth images, and its visual representation is tightly coupled with the specific robot's dynamics. As a result, many components must be retrained when switching to a new quadruped platform, which limits its ability to generalize rapidly across different morphologies.

In contrast, our proposed MGDP framework comprehensively overcomes these limitations. MGDP employs a U‐Net–based encoder to extract discriminative terrain features and leverages NVIDIA Warp for efficient large‐scale training, substantially reducing memory usage. Enabled by terrain‐adaptive reward functions, MGDP acquires diverse complex behaviors (e.g., climbing, jumping, crawling) in a single training stage, eliminating the need for cumbersome multi‐step distillation pipelines. Most importantly, the perception module is decoupled from robot dynamics, enabling the pre‐trained camera encoder to transfer across different quadruped platforms with only minimal adjustment.

## 3 Method Overview

**FIGURE 1:**
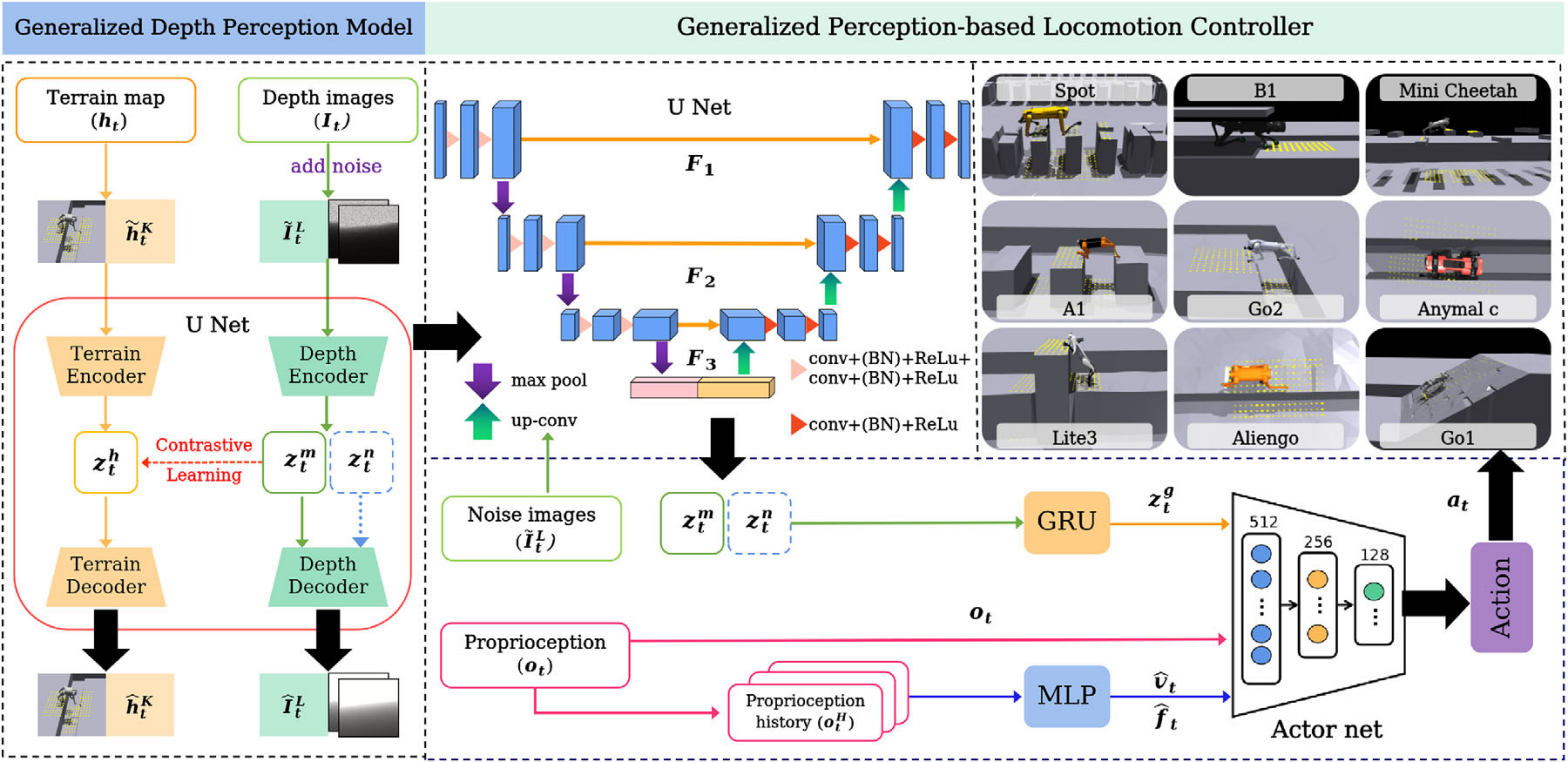
The framework of the proposed MGDP. The architecture is composed of a Generalized Depth Perception Model and a Generalized Perception‐Based Locomotion Controller. The perception model takes multi‐modal inputs (depth map and height map) and extracts a highly generalized, low‐dimensional terrain feature via contrastive learning. This model integrates an explicit depth map denoising mechanism and achieves an effective decoupling of terrain perception from robot dynamics. The locomotion controller combines this generalized feature with proprioceptive history to output the final joint torque commands. This decoupled design is critical for achieving the policy's superior cross‐terrain generality and fast platform transferability.

To address the limitations of existing perception‐based quadruped locomotion control methods, such as poor generalization in unstructured terrains, insufficient robustness to noise interference, and difficulty adapting to robots with different morphologies, this paper proposes the Mastering a Generalized Depth Perception Model (MGDP) framework. As shown in Figure 1, this framework comprises two core modules: the Generalized Depth Perception Model and the Generalized Perception‐based Locomotion Controller. These two modules work synergistically to enable robust, terrain‐aware locomotion of quadruped robots across diverse morphologies and complex unstructured environments. The former is designed to process noisy multi‐modal perceptual inputs (e.g., depth images and terrain maps) and to extract discriminative, noise‐resistant terrain feature representations via contrastive learning and an encoder‐decoder architecture, providing a reliable perceptual foundation for subsequent locomotion control. The latter aims to fuse the terrain features output by the perception module with the robot's proprioceptive data (e.g., joint states and inertial measurements), generate an unified adaptive locomotion policy through Proximal Policy Optimization (PPO) [20] and the Terrain‐Adaptive Reward Functions, enabling real‐time adjustment of locomotion to match diverse robot morphologies and terrain conditions, without multi‐stage skill distillation.

## 4 Generalized Depth Perception Model

Our Generalized Depth Perception Model (MGDP) aims to develop a generalized, efficient, and noise‐resistant terrain feature extraction mechanism, fundamentally addressing the core challenges faced by existing depth‐based perception controllers: high computational overhead, poor robustness to sensor noise, and deep coupling between perception and robot dynamics. First, the model adopts a multi‐modal input fusion, synchronously integrating depth images (for fine‐grained geometric details) and terrain height maps (for global elevation structure) to achieve comprehensive terrain characterization. Second, it leverages a U‐Net‐based multi‐encoder‐decoder architecture with skip connections to preserve multi‐scale terrain features effectively. Crucially, we integrate contrastive learning with dual reconstruction losses: by aligning latent embeddings of different modalities to enhance feature discriminability, while forcing the model to learn noise‐resistant representations through reconstructing clean data from noisy inputs. Additionally, to ensure training efficiency, we have built an optimized GPU acceleration mechanism based on NVIDIA Warp, supporting large‐scale parallel training of robots. Below, we will elaborate on the implementation details of the model.

### 4.1 Multi‐Modal Data Collection and Preprocessing

To provide high‐quality, task‐aligned training data for the Generalized Depth Perception Model while ensuring both data diversity and acquisition efficiency, we first synchronously collect multi‐modal perceptual data (terrain height maps and depth images) in the Isaac Gym simulation environment. This data is obtained from a variety of procedurally generated terrains, including slopes, stairs, obstacles, and rough uneven surfaces [21]. The subsequent preprocessing of the raw image data is crucial for robust feature extraction and model training.

#### 4.1.1 Data Collection

The built‐in camera of Isaac Gym incurs significant computational overhead and delays during large‐scale data collection, especially when generating parallel data for 4096 quadruped robots. The root cause is that the built‐in camera relies on the native rendering pipeline with redundant graphical computations (e.g., unnecessary texture rendering and lighting calculations) that are irrelevant to depth image acquisition. To address this, we developed a lightweight camera module by NVIDIA Warp [18]. Leveraging GPU‐accelerated warp operations, this module directly interacts with Isaac Gym's terrain physics engine to extract depth information, bypassing the redundant steps of the built‐in camera's rendering pipeline. This design not only reduces computational overhead but also boosts data acquisition speed compared to the built‐in depth camera. The parallel generation of raw depth images $I_{t}\in R^{B\times 120\times 120}$ can capture fine‐grained terrain details (such as gap edges, stone textures, and beam surfaces). Synchronously, we extract terrain height maps $h_{t}\in R^{B\times 187}$ directly from Isaac Gym. These maps follow a defined spatial range (aligned with the robot's local coordinate system): forward‐backward direction from –0.40 to 1.30 m, left‐right direction from –0.50 to 0.50 m, with a 0.1 m uniform sampling step for both axes. These depth images and terrain height maps form the multi‐modal perceptual data foundation for subsequent model training.

#### 4.1.2 Data Preprocessing

The raw depth images $I_{t}\in R^{120\times 120}$ undergo sequential processing via the preprocessing pipeline, including geometric adjustment, noise injection, normalization, and temporal buffering, to obtain the final output $I_{t}^{L}\in R^{1\times 2\times 16\times 16}$. The detailed steps are as follows.

The first step involves image cropping and resizing. For cropping, abnormal pixels in the original image are filtered out by clamping pixel values to preset bounds, expressed as:

(1) $$I_{t}^{clip}=clamp\left(I_{t},clip_{min},clip_{max}\right)\in R^{120\times 120}$$

where $clip_{min}=0.1m$ and $clip_{max}=3m$ are the preset bounds that limit the image's pixel values within a valid range. For resizing, the cropped image undergoes bilinear interpolation to adjust its resolution to $I_{t}^{resize}\in R^{16\times 16}$. Subsequently, dual noise injection is performed on the resized image to simulate the noise of real sensor: the process first adds Gaussian noise ($\varepsilon ∼N(0,\sigma^{2})$, with σ denoting the noise intensity) to model random measurement errors (avoiding noise amplitude being proportional to the original depth value). Then, we apply dropout noise to mimic sensor blind areas. Specifically, a binary mask tensor $M\in {\left\{0,1\right\}}^{16\times 16}$ and each element $M_{i,j}$ of the tensor follows a Bernoulli distribution. The final noisy image $I_{t}^{noisy}$ is expressed as:

(2) $$I_{t}^{noisy}=\left(I_{t}^{resize}+\varepsilon \right)⊙M\in R^{16\times 16}$$

where ⊙ denotes element‐wise multiplication. Next, we normalize the clean image and the noisy image separately, with both mapped to the [0,1] interval. This separate normalization strategy can avoid mutual interference between their pixel value distributions, as the clean image and noisy image may have different original value spans. The specific normalization formulas are as follows:

(3) $${\tilde{I}}_{t}^{clean}=\frac{I_{t}^{resize}-I_{\min}^{resize}}{I_{\max}^{resize}-I_{\min}^{resize}};{\tilde{I}}_{t}^{noisy}=\frac{I_{t}^{noisy}-I_{\min}^{noisy}}{I_{\max}^{noisy}-I_{\min}^{noisy}}$$

where $I_{min}$/$I_{max}$ are the original minimum/maximum values of the current frame image. Finally, sliding‐window temporal buffering is implemented to construct the temporal dimension of the output, with a temporal window length of L=2 for storing image frames. When a new image is added, the buffer updates by retaining the last L−1 frame and concatenating the noise frames ${\tilde{I}}_{t}^{L}\in R^{B\times 2\times 16\times 16}$ and clean frames $I_{t}^{L}\in R^{B\times 2\times 16\times 16}$, where B denotes the batch size. Separately, the height maps $h_{t}\in R^{17\times 11}$ are reshaped into $h_{t}^{K}\in R^{1\times 1\times 17\times 11}$, where K=1 denotes the single input channel.

### 4.2 Neural Network Design

To extract discriminative terrain representations from multimodal inputs, we adopt a U‐Net‐based multi‐encoder‐decoder architecture designed for depth images and terrain height maps, which consists of four modules: a Terrain Encoder, Terrain Decoder, Depth Encoder, and Depth Decoder. The two encoders share a common backbone and differ solely in input channel configuration and latent dimensionality, while the decoders are symmetric to their respective encoders, adhering to the inherent symmetric architecture of the U‐Net. In addition, skip connections are incorporated to merge multi‐scale features from the encoding stages into the decoder, mitigating information loss during downsampling and enabling the recovery of fine‐grained structural details. This design allows the network to capture both local geometric cues and global terrain structure, providing a robust representation for downstream tasks such as terrain perception and legged locomotion.

#### 4.2.1 Encoder Architecture

The Terrain Encoder and Depth Encoder adopt a three‐stage hierarchical downsampling architecture, where each stage is denoted $\Theta_{i},i\in \left\{1,2,3\right\}$. Each stage consists of two successive convolutions followed by Batch Normalization and ReLU activation, ensuring stable optimization and effective local feature extraction. The three stages progressively capture terrain information at different levels of abstraction. Specifically, the first stage focuses on local geometric cues such as edges and fine textures; the second stage aggregates mid‐level structural patterns, and the third stage encodes high‐level semantic representations without further spatial downsampling. Formally, the feature extraction process is expressed as:

(4) $$F_{1}=\Theta_{1}\left(X\right),F_{2}=\Theta_{2}\left(F_{1}\right),F_{3}=\Theta_{3}\left(F_{2}\right).$$

where $F_{1}$ captures fine‐grained geometric cues such as edges and local textures, $F_{2}$ aggregates mid‐level structural patterns, and $F_{3}$ extracts the deepest feature that preserves essential spatial information. Together, these three feature maps constitute the multiscale skip‐connection set:

(5) $$\mathrm{Skip}=\{F_{1},\;F_{2},\;F_{3}\},$$

By providing spatially aligned information for cross‐scale feature fusion, this set effectively improves decoding accuracy and preserves fine details. While the two encoders share the same convolutional backbone and three‐stage downsampling structure, they differ in their input configuration, latent projection design, and output activation. Specifically, the Terrain Encoder takes as input a single‐channel elevation map ${\tilde{h}}_{t}^{K}\in R^{1\times 1\times 17\times 11}$, whereas the Depth Encoder processes a two‐channel depth image pair ${\tilde{I}}_{t}^{L}\in R^{1\times 2\times 16\times 16}$. After the shared hierarchical feature extraction, the deepest feature map $F_{3}$ is passed through a modality‐specific projection head consisting of adaptive average pooling and two fully connected layers, producing distinct latent embeddings:

(6) $$Z_{t}^{h}=f_{h}\left(F_{3}\right)\in R^{B\times 32},$$

(7) $$Z_{t}^{m},Z_{t}^{n}=f_{i}\left(F_{3}\right)\in R^{B\times 64}.$$

#### 4.2.2 Decoder Architecture

The Terrain Decoder and Depth Decoder follow a symmetric design with respect to their corresponding encoders, progressively restoring spatial resolution through three upsampling stages while incorporating multiscale skip connections. The decoding process begins by transforming the latent vector $Z_{t}$, which may correspond to the height embedding $Z_{t}^{h}$ or the depth embeddings $\left(Z_{t}^{m},Z_{t}^{n}\right)$, into an initial low‐resolution feature map. This reshaped representation serves as the starting point for the subsequent hierarchical reconstruction.

At each decoding stage, the current feature maps are fused with the corresponding skip‐connection feature after spatial alignment using adaptive average pooling. The aligned features are concatenated along the channel dimension and passed through a transposed‐convolution block, followed by Batch Normalization and ReLU activation, which is written as:

(8) $$G_{1}=\Psi \left(Z,F_{3}\right),G_{2}=\Psi \left(G_{1},F_{2}\right),G_{3}=\Psi \left(G_{2},F_{1}\right),$$

where $G_{k}$ denotes the decoder feature map at the k‐th upsampling stage, and $F_{1}$, $F_{2}$, and $F_{3}$ are the shallow, middle, and depth encoder skip features, respectively. The operator Ψ(·) integrates encoder skip information with the current decoder state and refines the fused representation through deconvolution, enabling progressive spatial reconstruction.

Following the three‐stage upsampling process, the final decoder feature $G_{3}$ is mapped to the target spatial resolution through either bilinear interpolation or an additional transposed‐convolution layer, yielding the reconstructed output for each modality. A modality‐specific activation function is then applied to ensure numerical consistency with the physical meaning of the predicted signals:

(9) $$\begin{array}{cc} {\hat{h}}_{t}^{K} & =\tanh\left(G_{3}\right)\in R^{B\times 1\times 17\times 11}, \end{array}$$

(10) $$\begin{array}{cc} {\hat{I}}_{t}^{L} & =\sigma \left(G_{3}\right)\in R^{B\times 2\times 16\times 16}, \end{array}$$

where the Terrain Decoder produces a single‐channel height map, and the Depth Decoder generates a two‐channel depth image pair. Furthermore, the Terrain decoder applies a Tanh activation (tanh) to ensure compatibility with the normalized elevation representation, whereas the Depth decoder employs a Sigmoid activation (σ) to preserve the bounded nature of depth values. Other than their output activations and channel dimensions, the two decoders are architecturally identical.

### 4.3 Contrastive Learning With Reconstruction

Contrastive learning is a powerful self‐supervised paradigm, widely utilized in representation learning, that operates by attracting similar data points and repelling dissimilar ones in the latent space [22, 23]. In recent years, this paradigm has been increasingly adopted in robotic locomotion to enhance robustness and sim‐to‐real transfer. For instance, Mousa et al. [24] use contrastive learning to align student and teacher latent representations; Fu et al. [25] and Lu et al. [26] improve sim‐to‐real robustness via contrastive alignment between privileged and observable states. Furthermore, MOVE [27] applies a contrastive consistency objective to link different viewpoints and temporal segments for stable multi‐skill locomotion, while Chen et al. [28] structure a controllable latent space for transferable skills in humanoid robots. These studies highlight the effectiveness of contrastive learning in improving representation robustness, which motivates our incorporation of a contrastive objective in our framework.

Specifically, to jointly learn modality‐consistent and accurate spatial terrain reconstructions, we employ a hybrid loss function that integrates depth and height reconstruction objectives with a contrastive alignment term. Let ${\hat{I}}_{t}^{L}$ and ${\hat{h}}_{t}^{K}$ denote the reconstructed depth images and height map, and $I_{t}^{L}$ and $h_{t}^{K}$ their ground‐truth counterparts. The reconstruction losses are defined as mean squared error (MSE):

(11) $$\begin{array}{c} L_{\mathrm{depth}}=\frac{1}{N}\sum_{p=1}^{N}{\left({\hat{I}}_{t}^{L}\left(p\right)-I_{t}^{L}\left(p\right)\right)}^{2}, \end{array}$$

(12) $$\begin{array}{c} L_{\mathrm{height}}=\frac{1}{M}\sum_{q=1}^{M}{\left({\hat{h}}_{t}^{K}\left(q\right)-h_{t}^{K}\left(q\right)\right)}^{2}, \end{array}$$

where p and q index individual pixel locations in the depth and height domains, respectively, with N and M denoting the total number of elements (pixels × channels) in each map. It is important to note that the depth reconstruction loss compares the predicted clean depth images ${\hat{I}}_{t}^{L}$ against the clean ground‐truth depth $I_{t}^{L}$, rather than the noisy input observations. This ensures the model learns to denoise and correct sensor artifacts rather than merely reproducing them. To enforce cross‐modal alignment between height and depth embeddings, we introduce a contrastive loss to align the latent vectors $Z_{t}^{h}$ and $Z_{t}^{m}$. It encourages embeddings from the same timestep to remain close while separating mismatched pairs:

(13) $$L_{\mathrm{contrastive}}=-\log\frac{\exp\left(\mathrm{sim}(Z_{t}^{h},Z_{t}^{m})/\tau \right)}{\sum_{Z^{-}\in N_{t}}\exp\left(\mathrm{sim}(Z_{t}^{h},Z^{-})/\tau \right)},$$

where sim() and τ denote cosine similarity and temperature hyperparameter, $\left(Z_{t}^{h},Z_{t}^{m}\right)$ constitute the positive pair from the same timestep, and $N_{t}$ is the set of negative latent samples (denoted as $Z^{-}$) drawn from mismatched timesteps or modalities. The overall training objective is the weighted sum of these three losses:

(14) $$L_{\mathrm{total}}=L_{\mathrm{depth}}+L_{\mathrm{height}}+0.3\cdot L_{\mathrm{contrastive}}.$$

This unified formulation simultaneously enhances reconstruction fidelity and promotes cross‐modal embedding alignment, yielding terrain representations that are both geometrically accurate and semantically consistent.

## 5 Generalized Perception‐Based Locomotion Controller

We introduce a generalized perception‐driven locomotion controller that enables quadruped robots to traverse diverse terrains by leveraging the multi‐modal terrain representations provided by our perception model. The perception model encodes the terrain inputs into a latent space, allowing the locomotion controller to be trained or fine‐tuned across multiple robot morphologies without modifying the core perception architecture. The control problem is formulated as a Partially Observable Markov Decision Process (POMDP) [29]. In addition, we employ terrain‐adaptive reward functions and an asymmetric actor‐critic framework to learn a single expert policy capable of unified multi‐skill execution across different terrains, eliminating the need for multi‐stage skill distillation pipelines. The subsequent subsections provide a detailed description of these essential components.

### 5.1 Observations and Action Space

While a robot can acquire real‐time proprioceptive and exteroceptive sensory inputs, several key privileged states, such as body velocity, foot swing height, and terrain maps, are not directly observable in real‐world scenarios. Thus, the perception‐based locomotion problem can also be formulated as a POMDP, where the proprioceptive observation $o_{t}$ is written as: $o_{t}=\left(\omega_{t},\;g_{t},\;v_{t}^{*},\;q_{t},\;{\dot{q}}_{t},\;a_{t-1}\right)\in R^{45}$, where $\omega_{t}\in R^{3}$, $g_{t}\in R^{3}$, $v_{t}^{*}\in R^{3}$, $q_{t}\in R^{12}$, ${\dot{q}}_{t}\in R^{12}$, $a_{t-1}\in R^{12}$ denote body angular velocity, projected gravity, linear velocity command, joint angles, joint angular velocities, and the action of the last step. We further construct a temporal observation $o_{t}^{H}=\left[o_{t},o_{t-1},\text{…},o_{t-H}\right]$, which aggregates the proprioceptive observations from the past H=5 time steps.

The policy outputs a 12‐dimensional action vector $a_{t}\in R^{12}$, interpreted as a residual joint position update. The resulting target configuration is $q_{t}^{*}=q_{t}+a_{t}$, while the simulator's joint‐level PD controller handles torque generation, $\tau_{t}=K_{p}\cdot \left(q_{t}^{*}-q_{t}\right)+K_{d}\cdot \left(-{\dot{q}}_{t}\right)$, where $K_{p}$ and $K_{d}$ are diagonal proportional and derivative gain matrices, and ${\dot{q}}_{t}$ represents the measured joint velocity.

### 5.2 Terrain‐Adaptive Reward Functions

We adopt three types of fundamental reward components widely used in prior quadruped locomotion studies [6], [8], [21]: Tracking, which encourages accurate velocity following; Smoothness, which penalizes abrupt or energetically inefficient actions; and Safety, which discourages joint‐limit violations, stumbling, and unstable contacts. These baseline rewards, summarized in Table 1, provide essential locomotion stability and regularization. However, these generic rewards are insufficient for guiding reliable locomotion across diverse parkour terrains. To learn a unified policy, we introduce a set of terrain‐adaptive curriculum rewards that modulate their penalty strength according to the terrain class. This mechanism enables the policy to develop terrain‐specific behaviors, such as maintaining consistent body height in narrow corridors or performing larger body‐lifting motions over stepping stones.

**TABLE 1:** Reward Terms.

Terms	Reward	Equation	Weight
Tracking	Lin. velocity tracking	$e^{-4||v_{xy}^{*}-v_{xy}{\left|\right|}^{2}}$	1.0
	Ang. velocity tracking	$e^{-4{\left(w_{yaw}^{*}-w_{yaw}\right)}^{2}}$	0.5
Smoothness	Angular velocity (xy)	$\left|\right|w_{xy}{\left|\right|}^{2}$	−0.05
	Joint torque	${\left||\tau |\right|}^{2}$	$-e^{-5}$
	Action rate	$\left|\right|a_{t}-a_{t-1}{\left|\right|}^{2}$	−0.01
	Joint accelerations	$\left|\right|\ddot{q}{\left|\right|}^{2}$	$-2.5e^{-7}$
	Motion limit	$\left|\right|q_{\text{FL}}-q_{\text{RR}}{\left|\right|}_{1}+\left|\right|q_{\text{FR}}-q_{\text{RL}}{\left|\right|}_{1}$	−0.1
Safety	Joint pose limit	$\left||\max(0,q_{t}^{\min}-q_{t}\right)$ $+\max\left(0,q_{t}-q_{t}^{\max}\right){\left|\right|}_{1}$	−10
	Feet air time	$\sum_{i=0}^{4}\left(t_{air,f_{i}}-0.5\right)$	1.0
	Feet stumble	$\text{any}\left(∥f_{xy}∥>4\cdot |f_{z}|\right)$	−1.0
	Feet edge	$\sum_{i=0}^{4}I_{c}\cdot M\left[p_{f_{i}}\right]$	−1.0
Curriculum	Linear z velocity	Equation (15)	−1.0
	Orientation	Equation (17)	−0.2
	Collision	Equation (19)	−1.0

**FIGURE 2:**
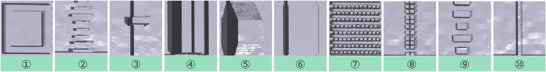
The ten different terrains in our task, including ① single‐gaps, ② air‐beams, ③ hurdles, ④ narrow corridors, ⑤ ramps, ⑥ air‐stones, ⑦ stones‐everywhere, ⑧ stones‐2rows, ⑨ continuous‐gaps, ⑩ single‐bridges.

To implement this, we first construct ten representative terrain classes in simulation, as shown in Figure 2, including single‐gaps, air‐beams, hurdles, narrow corridors, ramps, air‐stones, stones‐everywhere, stones‐2rows, continuous‐gaps, and single‐bridges. During training, terrain difficulty is increased by widening gaps, reducing beam widths, raising obstacle heights, or tightening corridor constraints.

Based on these terrains, we design three terrain‐adaptive reward terms “Linear z Velocity, Orientation, and Collision” that adjust their weight according to the current terrain class. The detailed formulations are presented below.

#### 5.2.1 Linear z Velocity Reward

To regulate unnecessary vertical oscillation while still allowing sufficient body lifting on terrains that require greater stepping height, we introduce a terrain‐adaptive penalty on the robot's vertical base velocity. This reward is defined as:

(15) $$r_{\text{lin-}z}\left(t\right)=-\;w_{z}\left(c\right)\;v_{z,t}^{2},$$

where $v_{z,t}$ denotes the vertical linear velocity of the base at time t, and c is the terrain class index. The terrain weight $w_{z}\left(c\right)$ adjusts the penalty strength according to terrain class:

(16) $$w_{z}\left(c\right)=\left\{\begin{array}{cc} 0.10, & c=②,\\ 0.01, & c=③,\\ 0.05, & c=\{①,⑦,⑨\},\\ 1.00, & \text{otherwise}. \end{array}\right.$$

#### 5.2.2 Orientation Reward

To maintain stable body posture when traversing terrains requiring precise balance or narrow‐footprint stepping, we introduce a terrain‐adaptive penalty on the robot's roll–pitch deviation from the upright orientation. The reward $r_{\text{orient}}\left(t\right)$ and terrain weight $w_{\text{orient}}\left(c\right)$ are defined as:

(17) $$r_{\text{orient}}\left(t\right)=-\;w_{\text{orient}}\left(c\right)\;{\left\|q_{xy,t}\right\|}^{2},$$

(18) $$w_{\text{orient}}\left(c\right)=\left\{\begin{array}{cc} 0.01, & c=\{②,③\},\\ 1.00, & \text{otherwise}. \end{array}\right.$$

where $q_{xy,t}$ denotes the roll and pitch components of the robot's orientation at time t.

#### 5.2.3 Collision Reward

To mitigate unsafe contacts with obstacles and ensure reliable locomotion in cluttered environments, we introduce a terrain‐adaptive collision penalty reward, which suppresses undesired impacts between the robot and the surrounding environment. The reward is defined as:

(19) $$r_{\text{collision}}\left(t\right)=-\;w_{\text{col}}\left(c\right)\;n_{\text{collision}}\left(t\right),$$

where $n_{\text{collision}}\left(t\right)$ denotes the number of collision events detected at time t. The terrain‐dependent coefficient $w_{\text{col}}\left(c\right)$ scales the penalty to reflect the risk severity:

(20) $$w_{\text{col}}\left(c\right)=\left\{\begin{array}{cc} 0.0, & c=④,\\ 0.20, & c=\{①,②,⑤\},\\ 10.0, & c=\{③,⑥\},\\ 1.00, & \text{otherwise}. \end{array}\right.$$

where $v_{xy}^{*}$, $v_{xy}$, $w_{yaw}^{*}$, and $w_{yaw}$ are the desired linear velocity, the actual linear velocity, the desired, and actual yaw angular velocity, respectively. $w_{xy}$ is the actual pitch and roll angular velocity in the xy plane. $\ddot{q}$ is the joint acceleration vector. $q_{\text{FL}}$, $q_{\text{RR}}$, $q_{\text{FR}}$, and $q_{\text{RL}}$ are the joint positions for the Front‐Left, Rear‐Right, Front‐Right, and Rear‐Left legs, respectively. $t_{air,f_{i}}$ is the time in air for foot $f_{i}$. $f_{xy}$ is the foot contact force vector in the xy plane (tangential force), and $f_{z}$ is the foot contact force in the z axis (normal force). $I_{c}$ is the contact indicator, which is 1 if in contact. M denotes a boolean function which is 1 if the point $p_{f_{i}}$ lies within 5 cm of an edge. $p_{f_{i}}$ denotes the foot position for each leg.

### 5.3 Asymmetric Actor‐Critic

To fully leverage the multimodal terrain representations produced by our perception model, we adopt an asymmetric actor–critic architecture for policy learning, as illustrated in Figure 1. The core idea is to exploit perceptual features and privileged information during training to improve sample efficiency and stabilize optimization, while ensuring that the learned policy remains deployable under real‐world sensing constraints. Benefiting from the generality of our perception model, the locomotion controller can be trained or fine‐tuned on nine quadruped platforms “including A1, Aliengo, Anymal C, B1, Go1, Go2, Lite3, Mini Cheetah, and Spot” without redesigning the perception pipeline. This enables rapid acquisition of parkour locomotion across diverse robot embodiments.

During training, the perception module provides two instantaneous visual embeddings, $z_{t}^{m}$ encoding height‐related features and $z_{t}^{n}$ capturing depth appearance features. To obtain a stable and history‐aware representation, they are passed through a GRU that aggregates temporal features:

(21) $$z_{t}^{g}=\mathrm{GRU}\;\left(\left[z_{t}^{m},\;z_{t}^{n}\right],\;z_{t-1}^{g}\right)\in R^{64},$$

where $z_{t}^{g}$ denotes the temporally smoothed terrain feature. In addition to visual perception, the actor requires estimates of several quantities that are not directly observable on real robots, such as the body linear velocity and the foot swing height. To infer these latent states, we employ an estimator (MLP) that processes the history of proprioceptive observations $o_{t}^{H}$ and produces their estimates:

(22) $${\hat{v}}_{t},\;{\hat{f}}_{t}=\mathrm{MLP}\;\left(o_{t}^{H}\right)\in R^{7},$$

where ${\hat{v}}_{t}$ represents the estimated body velocity and ${\hat{f}}_{t}$ the estimated foot height. Combining all deployable information, the input to the actor network is defined as

(23) $$p_{t}=\left[o_{t},\;{\hat{v}}_{t},\;{\hat{f}}_{t},\;z_{t}^{g}\right]\in R^{116}.$$

In addition to the proprioceptive observation, the critic further receives privileged information that exists only in simulation, including the body velocity $v_{t}$, foot heights $f_{t}$, and the height map $h_{t}$. The full critic input is written as:

(24) $$s_{t}=\left(o_{t},v_{t},f_{t},h_{t}\right)\in R^{239}.$$

## 6 Experimental Setting

### 6.1 Simulation

We begin by leveraging the Isaac Gym simulator [30] to train 4096 parallel Unitree Go2 robots on conventional challenging terrains, combined with extensive domain randomization as summarized in Table 2. Each episode is terminated and reset when either (i) a collision occurs between the robot body and the terrain, or (ii) the robot becomes immobilized for more than 20 seconds. Importantly, our perception model is trained jointly during this stage, allowing it to acquire terrain‐understanding capabilities that are naturally aligned with the locomotion training context.

**TABLE 2:** The Randomization range of parameters.

Parameters	Range	Unit
$K_{p}$ factor	[0.9, 1.1]	Nms/rad
$K_{d}$ factor	[0.9, 11]	Nms/rad
Payload	[–1.0, 2.0)	kg
System delay	[0.0, 12]	ms
Frictions coefficient	[0.2, 1.25)	—
Center of mass shift	[–0.05, 0.05)	m
Motor strength factor	[0.9, 1.1]	Nm
Noise of terrain map $h_{t}$	[–5, 5)	cm
Noise of camera pitch	[–2, 2]	deg
Noise of camera position C	[–2, 2]	cm

After the base controller converges, we further fine‐tune both the locomotion policy and the perception module on a collection of newly designed parkour‐style terrains, as shown in Figure 2. When training diverse parkour skills, the perception model is updated together with the locomotion policy, thereby improving its adaptability and enriching the terrain data it has seen. This joint fine‐tuning enables the robot to develop more advanced gait adaptation strategies tailored to diverse and high‐risk environments, significantly improving its robustness in parkour terrains. During fine‐tuning, any robot in Table 3 (e.g., A1, Go1, Spot, Anymal C) can be used, and adapting to a new platform requires only adjusting the camera pose, as well as the corresponding stiffness and damping parameters. The fine‐tuning process does not require any additional changes to the training framework, and typically, approximately 10 000 episodes (on the order of 14.5 h in total) are sufficient to seamlessly transfer our perception–control pipeline across diverse quadruped morphologies.

**TABLE 3:** The Randomization range of different robots.

Robots	Camera position (C)	$K_{p}$ (Stiffness)	$K_{d}$ (Damping)
A1	[0.30, 0, 0.04]	30	0.8
B1	[0.48, 0, 0.10]	80	2.0
Go1	[0.30, 0, 0.07]	30	0.8
Go2	[0.34, 0, 0.06]	30	0.8
Lite3	[0.29, 0, 0.06]	20	0.6
Spot	[0.52, 0, 0.10]	60	1.8
Aliengo	[0.35, 0, 0.04]	40	1.2
ANYmal C	[0.52, 0, 0.10]	80	1.2
Mini Cheetah	[0.26, 0, 0.05]	20	0.6

The perception model consists of two convolutional encoders for depth images and height maps, using channel configurations of [32,64,64] and [16,32,64], respectively, each paired with a symmetric decoder that reconstructs its corresponding modality. The two visual embeddings generated by the encoders are fused through a GRU with a 64‐dimensional hidden state, which is optimized together with the control policy during training. Similar to the architecture described in [5, 8], the control network utilizes a Multi‐Layer Perceptron (MLP) to process proprioceptive history. This MLP consists of hidden layers with sizes [256,128,7]. The entire training is performed on a desktop PC with Intel (R) Gold 6226R CPU @ 2.90 GHz, 32 GB RAM, and an NVIDIA RTX 3090 GPU. Training the perception model and the perception controller costs approximately 8 and 14 h, respectively.

### 6.2 Hardware

The MGDP system is deployed on a Unitree Go2 quadruped robot equipped with an Intel RealSense D435 depth camera operating at 60 Hz. A control loop running at 50 Hz synchronizes the incoming depth frames with the policy inference, ensuring consistent timing between perception and locomotion. All computation, including depth processing and policy execution, is performed on an external laptop connected to the robot via Ethernet, using an Intel i7‐12700H CPU for real‐time operation. The robot is controlled through joint‐space PD gains of $k_{p}=30$ and $k_{d}=0.8$, which provide stable tracking performance across diverse terrains.

## 7 Results and Discussion

### 7.1 Comparison with Blind‐Based Quadruped Controllers

**FIGURE 3:**
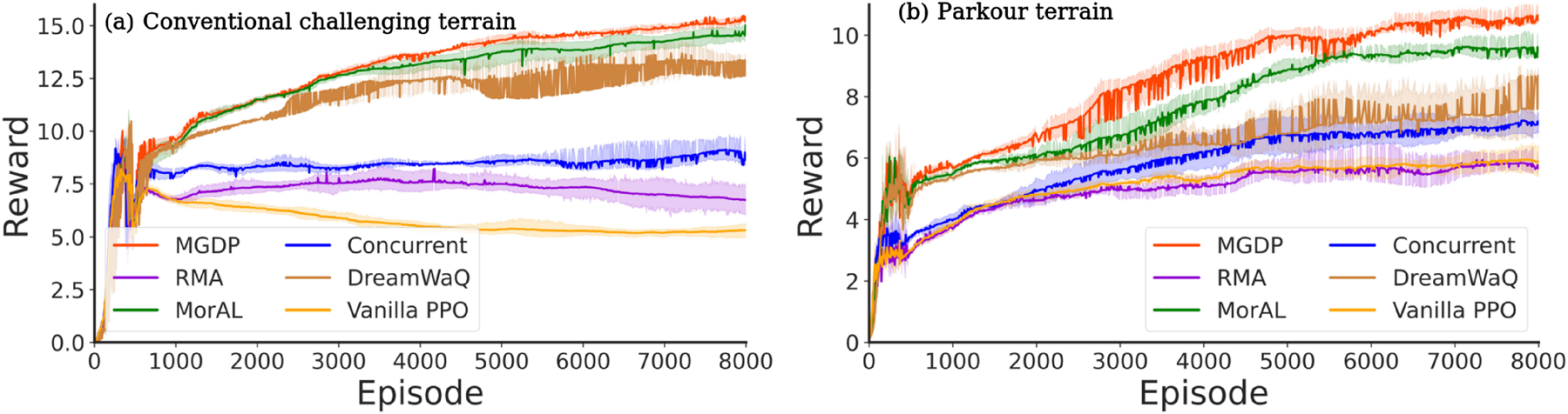
The average rewards of six blind controllers on 4096 Go2 over 6000 episodes for two different types of terrain: (a) Parkour terrain and (b) Conventional challenging terrain. Data presented as mean ± standard deviation; n=5 independent random seeds per method. Each curve's shaded region represents the standard deviation.

Figure 3 depicts the average episode rewards of our MGDP in comparison with five blind‐based controllers (MorAL [6], Concurrent [16], RMA [15], DreamWaQ [6], and Vanilla PPO [21]) trained from scratch over 8,000 episodes on two distinct terrain categories: (a) Conventional Challenging terrain, and (b) Parkour terrain. In Conventional Challenging terrain, MGDP demonstrates the most robust and stable learning performance and consistently achieves the highest average rewards among all comparator methods throughout training (Figure 3a). MorAL and DreamWaQ show significant late‐stage improvements but ultimately yield lower final rewards than MGDP. While Concurrent and RMA achieve moderate rewards, their learning curves exhibit substantially high‐frequency fluctuations, indicative of less stable policy development. Vanilla PPO consistently yields the lowest performance. The performance gap between MGDP and MorAL remains small, which is primarily because the difficulty in terrains such as uneven surfaces and slopes stems mainly from mastering the robot's internal dynamics. Thus, most blind algorithms can adapt using proprioceptive feedback, diminishing the necessity for proactive environmental perception in these terrains.

In the Parkour terrain, the performance disparity among the algorithms becomes profoundly more pronounced. MGDP demonstrates an overwhelming superiority, which is an order of magnitude higher than its competitors. This performance leap underscores the critical advantage conferred by its environmental perception capability. MorAL shows a steadily increasing trend, reaching a distant second place, while DreamWaQ's performance remains considerably lower. Concurrent and RMA exhibit moderate, highly volatile learning curves, failing to adapt effectively to the terrain's demands. Vanilla PPO consistently yields negligible rewards, indicating a near‐complete failure to learn a viable policy. The dramatic performance gap highlights that the key challenge of the parkour terrain is insurmountable without proactive environmental perception. Unlike methods relying on reactive proprioceptive strategies, MGDP's ability to anticipate and plan around discrete obstacles is indispensable, transforming a prohibitively difficult task into a solvable one.

### 7.2 Comparison With Perception‐Based Quadruped Controllers

To validate the effectiveness and generalized competence of the MGDP, we provide a detailed comparative analysis against the following state‐of‐the‐art perception‐based controllers.

- WMP [19]: This method predicts future latent visual–geometric states through a recurrent world model, allowing the controller to remain stable under noisy or delayed perception. By providing a temporally consistent latent representation, WMP improves robustness on dynamic and visually challenging terrains.
- PIE [11]: This policy predicts latent body dynamics and explicit terrain cues from depth and proprioceptive inputs, enabling quadrupedal robots to perform robust and agile parkour maneuvers on challenging obstacles.
- Parkour [12]: This approach trains a single end‐to‐end vision‐based policy that enables quadrupedal robots to perform diverse parkour skills, including climbing, leaping, crawling, and squeezing. This approach adopts a two‐stage DRL paradigm, enabling the robot to acquire a unified parkour policy via skill distillation.
- Extreme Parkour [4]: This method trains a single end‐to‐end policy that maps depth images from a low‐cost onboard camera directly to motor commands via DRL, which enables the quadruped to perform extreme parkour skills.
- MARG [8]: This policy utilizes a specialized framework to safely cross different risky gap terrains, relying on elevation mapping for precise foothold selection.
- Multi‐Layer Parkour [9]: This method leverages multi‐layer elevation maps for precise perception of complex multi‐tiered terrains and adopts skill distillation to integrate specialized traversal skills, enabling autonomous and safe quadruped traversal across multi‐layer scenarios.
- Anymal Parkour [17]: This policy is specialized for the ANYmal quadrupedal robot, focusing on agile navigation by integrating proprioceptive and exteroceptive inputs (e.g., LiDAR, depth camera) to enable aggressive maneuvers, such as gap clearance, jumping, and stable landing across complex parkour terrains.

Table 4 presents a comparative analysis of computational resources and training complexity across several state‐of‐the‐art methods. The proposed MGDP controller demonstrates highly competitive performance coupled with superior efficiency. Crucially, it employs a single‐phase training pipeline, thereby eliminating the complex multi‐stage distillation required by methods like Parkour, Extreme Parkour, and Multi‐Layer Parkour. Architecturally, MGDP operates with a lower GPU memory footprint (e.g., versus ≈20 GB for World Model, >20 GB for PIE, and >45 GB for Anymal Parkour) while still supporting large‐scale training with 4096 parallel agents. Furthermore, MGDP achieves robust parkour locomotion using only one depth camera, successfully avoiding the hardware complexity and cost associated with multi‐sensor setups.

**TABLE 4:** Comparison with different perception‐based quadrupedal locomotion controllers.

Controllers	Agent numbers	GPU memory	Each iteration time	Distillation	Extra sensors
MGDP (This work)	4096	≈ 12 GB	≈ 7.0 s	No	1 Depth Camera
WMP [19]	4096	> 20 GB	≈ 10.7 s	No	
PIE [11]	4096	> 20 GB	≈ 7.2 s	No	
Parkour [12]	256	≈ 19.0 GB	≈ 2.1 s	Yes	
Extreme Parkour [4]	192	≈ 14.5 GB	≈ 5.7 s	Yes	
MARG [8]	4096	≈ 8 GB	≈ 3.8 s	No	1 LiDAR
Multi‐Layer Parkour [9]	4096	≈ 10 GB	≈ 4.5 s	Yes	
Anymal Parkour [17]	4096	> 45 GB	None	Yes	1 Depth Camera and 6 LiDARs

In contrast, while MARG also benefits from low GPU memory consumption and single‐phase training, its approach relies on a LiDAR‐based elevation map. This method not only demands an accurate terrain map generation model with high parameter tuning costs and inherently struggles to handle specific terrains, such as narrow gap traversal and under‐overhang maneuvers, but also poses significant challenges for fast transfer to new robot platforms.

### 7.3 Ablation of Depth Noise

To quantify the impact of the denoising module on locomotion performance, we simulate depth noise under extreme real‐world conditions in simulation: Gaussian noise with standard deviation 0.06 and binary dropout with probability 0.2 are injected into the depth map (stronger than the training‐time setting to better assess controller robustness and generalization). Under this noise configuration, we evaluate four training conditions “without depth noise, with Gaussian noise only, with Dropout noise only, and with combined Gaussian+Dropout noise” on 10 Go2 robots across 10 terrain categories, with 10 traversal episodes per robot and terrain. For each episode we record four metrics: **failure rate (%)** (episode‐level failure proportion), **collision rate (%)** (step‐normalized collision proportion), **travel distance (m)** (mean horizontal path length per episode, from integrating $∥v_{xy}∥\cdot dt$ along the trajectory), and **tracking error (m/s)** (RMSE between commanded and actual xy velocity). We then aggregate these metrics over terrain difficulty levels 1–10 to obtain the quantitative comparison curves.

**FIGURE 4:**
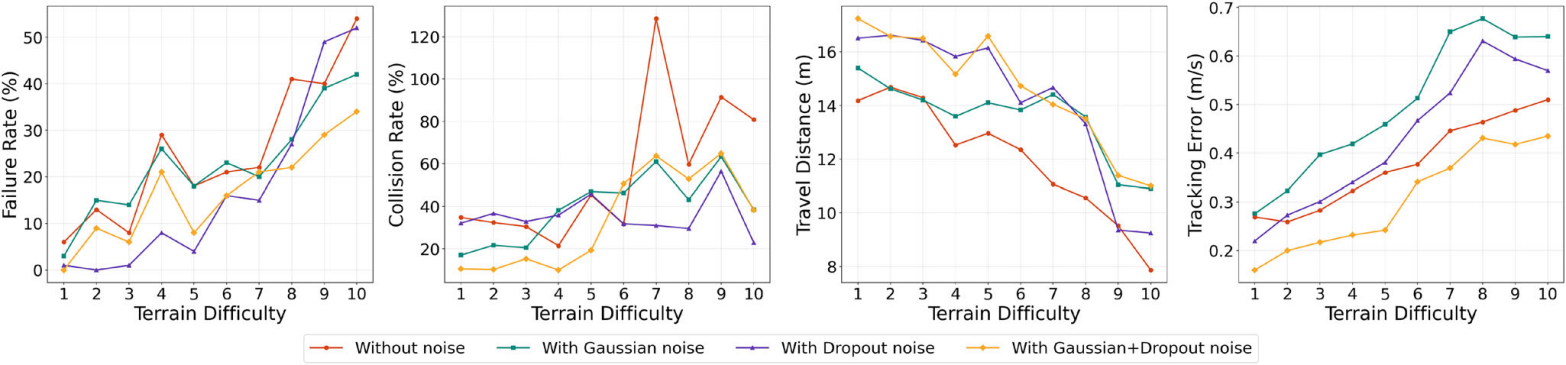
Ablation of depth noise on locomotion performance. The figure compares four training conditions (without noise, Gaussian, Dropout, Gaussian+Dropout) across 10 different terrain difficulty levels using four evaluation metrics: (a) failure rate (%), (b) collision rate (%), (c) travel distance (m), and (d) tracking error (m/s).

Figure 4 reports the four metrics across terrain difficulty levels 1–10. **(a)** Failure rate: all conditions show a rising failure rate with difficulty; the policy trained without noise exhibits the sharpest increase at greater difficulties, while the policy trained with Gaussian+Dropout noise maintains the lowest failure rate (e.g., below 35% at difficulty 10). **(b)** Collision rate: the without‐noise policy shows a sharp collision peak (e.g., exceeding 120% at difficulty 7); policies trained with noise (Gaussian, Dropout, or both) are more stable, and Gaussian+Dropout or Dropout alone achieve lower collision rates at higher difficulties. **(c)** Travel distance: travel distance decreases with difficulty for all conditions; the without‐noise policy achieves the lowest values, while Gaussian+Dropout achieves the highest travel distance across most difficulties. **(d)** Tracking error: tracking error increases with difficulty; the Gaussian+Dropout policy maintains the lowest tracking error, and the Gaussian‐only policy shows the highest.

Overall, the four panels show that training with combined depth noise (Gaussian+Dropout) yields the most robust performance across all metrics: it attains the lowest failure rate and tracking error and the highest travel distance, while avoiding the severe collision peak seen with the without‐noise policy. Policies trained with a single noise type (Gaussian or Dropout) lie between these two extremes. These results support that the denoising module and noise‐informed training preserve locomotion performance under the tested noisy depth input and improve robustness and generalization.

### 7.4 Ablation of Terrain‐Adaptive Reward

**FIGURE 5:**
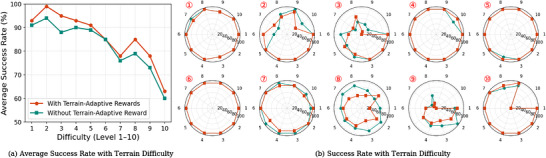
Ablation of terrain‐adaptive reward vs. fixed‐weight reward. (a) Success rate vs. terrain difficulty level (1–10). (b) Performance by terrain difficulty level (1)–(10), with vs. without terrain‐adaptive reward.

To validate the contribution of the terrain‐adaptive reward design, we compare two training conditions under the same setup: (i) with terrain‐adaptive rewards and (ii) without terrain‐adaptive rewards (fixed‐weight baseline). For each condition, we use 10 Go2 robots to evaluate traversal performance on 10 representative terrain categories, with each robot performing 10 traversal episodes per terrain. For every terrain and training condition, we compute both terrain‐wise and overall **success rate (%)**. In addition, we collect data separately for terrain difficulty levels 1–10. To reveal how the reward design affects difficulty‐dependent performance, we plot success‐rate curves as a function of terrain difficulty and present radar charts organized by terrain difficulty across different dimensions, as shown in Figure 5.

Figure 5 reports these results. **(a)** Success rate versus terrain difficulty level (1–10): the terrain‐adaptive reward consistently maintains a higher success rate than the fixed‐weight variant as difficulty increases; the gap widens at higher difficulties, indicating that the adaptive design is especially beneficial on harder terrains. **(b)** Radar charts for the ten terrain difficulty levels (1)–(10), each showing performance across dimensions under the two reward settings: the policy with terrain‐adaptive reward (orange) generally achieves equal or higher values than the fixed‐weight variant (teal) across difficulty levels, demonstrating that the adaptive design adds clear value in task completion and robustness.

Overall, these results show that the terrain‐adaptive reward functions enable the controller to sustain higher success rates and reduce safety‐critical events relative to the fixed‐weight baseline, especially on challenging terrains. This validates that explicitly encoding terrain‐dependent rewards is an effective way to improve both robustness and overall task performance.

### 7.5 Evaluation of Terrain Traversal Capability

**FIGURE 6:**
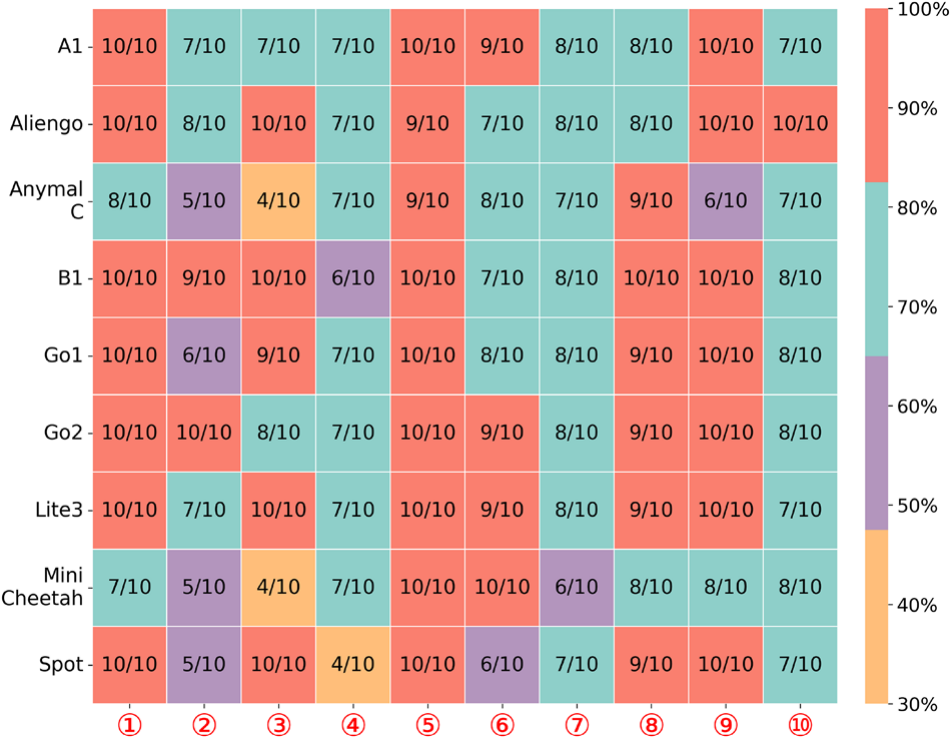
Comparison of terrain traversal performance among 9 quadruped robots across 10 types of extreme terrains. Each cell displays the ratio of the maximum difficulty that a robot can stably traverse to the greatest difficulty of the corresponding extreme terrain.

We further assess the terrain traversal capability of 9 mainstream quadruped robots. As shown in Figure 6, it intuitively illustrates their core performance across 10 types of extreme terrains (detailed in Figure 2). Each terrain is configured with 10 difficulty levels (ranging from 1 to 10, with 10 denoting the greatest difficulty). The ratio formatted as maximum traversable difficulty / maximum terrain difficulty in each cell serves as the core metric for quantifying the upper limit of the robot's traversal capability. A ratio closer to 1 indicates that the maximum difficulty the robot can stably traverse is nearer to the terrain's upper limit, thereby reflecting superior terrain adaptability. The results demonstrate the robust and generalized nature of the MGDP policy, achieving high traversal success rates across the majority of the tested robot‐terrain combinations. Specifically, Go2 and Aliengo demonstrate exceptional adaptability to complex and challenging environmental conditions, maintaining ratios of 0.8 or higher in most terrains. In contrast, Anymal C and Mini Cheetah exhibit relatively limited adaptability, with ratios below 0.5 on ③ (hurdles) and ④ (narrow corridors), suggesting they can only handle low‐difficulty levels (≤ Level 4) of these two terrains. This discrepancy can be primarily attributed to the inherent locomotion constraints of the robots' own configurations: for instance, Mini Cheetah's smaller body size restricts its stride length and obstacle‐crossing capability, while Anymal C's larger body and initial posture calibration differences reduce its maneuverability in narrow or spatially constrained terrains. Despite these platform‐specific limitations, most platforms (especially B1, Spot, Go2, and Aliengo) achieve high success ratios on ① (single‐gaps), ⑤ (ramps), ⑧ (stones‐2rows), and ⑨ (continuous‐gaps). Overall, the single MGDP policy successfully transfers multi‐skill locomotion capabilities to a wide array of robotic platforms, achieving robust control with high performance consistency.

### 7.6 Analyzing the Loss of the Perception Model

**FIGURE 7:**
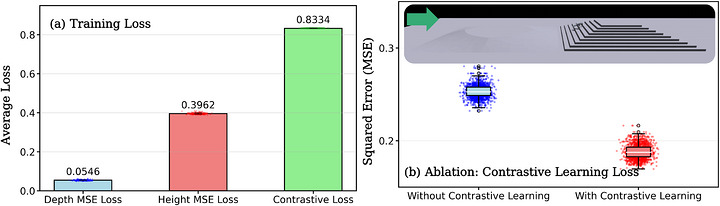
Analysis of training loss and ablation contrastive learning for the perception model. (a) Training losses (Depth MSE, Height MSE, Contrastive Loss) during Episodes 6000–8000; data presented as mean over the training batch. (b) Ablation of Contrastive Learning via latent feature MSE.

Figure 7(a) displays three core loss metrics (Depth MSE Loss, Height MSE Loss, and Contrastive Loss) analyzed during the model's stable training phase (Episodes 6000–8000), where they all reach low, stable values averaging 0.0546, 0.3962, and 0.8334, respectively. The low Depth MSE Loss indicates high‐fidelity depth image reconstruction with minimal loss of fine‐grained details like obstacle edges and terrain textures; the stable Height MSE Loss verifies reliable terrain height map estimation for accurate elevation references in quadruped foot placement planning; and the reasonable Contrastive Loss value ensures the model learns discriminative features that distinguish terrain types, indirectly enhancing the semantic relevance of visual features to terrain elevation and structure. Collectively, these results demonstrate the model's excellent convergence, high‐precision terrain perception reconstruction, and stability, laying a reliable perceptual foundation for quadruped robots to navigate complex terrains.

To evaluate the effectiveness of contrastive learning, Figure 7b presents an ablation study comparing the MSE of latent features with and without Contrastive Learning. The results show that the model with Contrastive Learning achieves a significantly lower squared error, indicating that contrastive learning effectively enhances the discriminative power of latent features. This reduction in error demonstrates that the learned features are more robust and meaningful, enabling better terrain distinction and thus improving the overall perception capability for quadruped locomotion in complex environments.

### 7.7 Denoising and Reconstruction of the Perception Model

**FIGURE 8:**
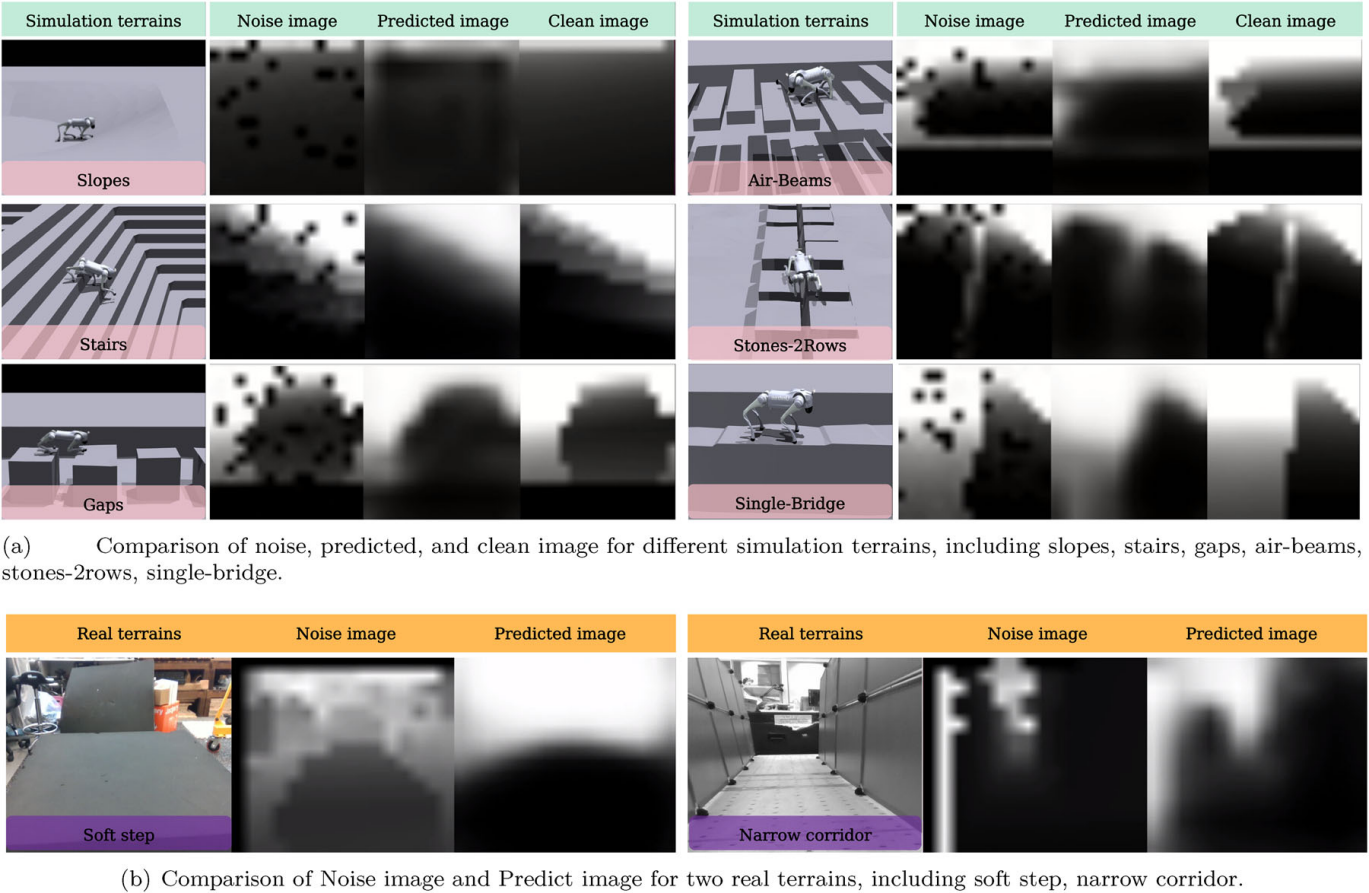
Image denoising and reconstruction results on simulation and real terrains.

To further evaluate the perception model's image denoising and reconstruction capability, Figure 8 presents the results on both simulation and real terrains. For simulation terrains including Slopes, Stairs, Gaps, Air‐Beams, Stones‐2Rows, and Single‐Bridge, Figure 8a compares the noise image (with artificial noise), predicted image (denoised by the model), and the clean image (ground truth without noise). The model demonstrates robust denoising performance, as predicted images closely match clean images, preserving key terrain details such as slope gradients, stair edges, and gap boundaries. For real terrains (Soft step and Narrow corridor), Figure 8b contrasts the noise image (real‐world noisy input) and the predicted image (model's output). Even with real‐world noise, the predicted images effectively recover terrain structures (e.g., the contour of the soft step and the layout of the narrow corridor), indicating the model's generalization ability to real environments. These results collectively validate that the perception model can reliably denoise and reconstruct terrain images, providing clear and accurate visual inputs for quadruped robots to perceive complex terrains.

**FIGURE 9:**
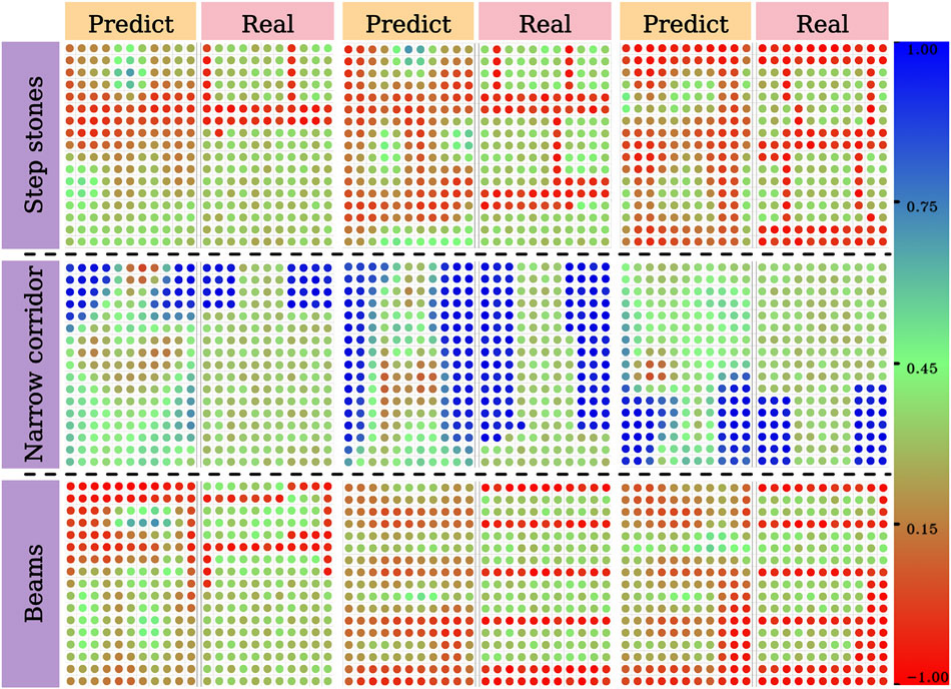
Comparison of predicted and real height maps across different terrains (step stones, narrow corridor, beams) at different time steps.

Figure 9 compares the predicted and real height maps across three terrains “Step stones, Narrow corridor, and Beams” at different time steps, with a color scale ranging from –1.00 (red) to 1.00 (blue).

#### 7.7.1 Step Stones

The predicted height maps closely align with the actual ones, exhibiting similar color distributions and indicating accurate elevation predictions over time.

#### 7.7.2 Narrow Corridors

Both predicted and real maps feature noticeable blue regions representing structural elevation, with slight differences in the extent of these regions pointing to minor prediction errors.

#### 7.7.3 Beams

The predicted and real maps demonstrate strong consistency, with most areas clustered in red and green, reflecting the model's reliable prediction of the terrain's relatively low and uniform elevation. Overall, the figure demonstrates that the model achieves good performance in predicting height maps across diverse terrains, with high fidelity in capturing height map patterns over time and providing rich information on terrain variation for legged locomotion.

### 7.8 Real‐World Transfer on Indoor Terrains

**FIGURE 10:**
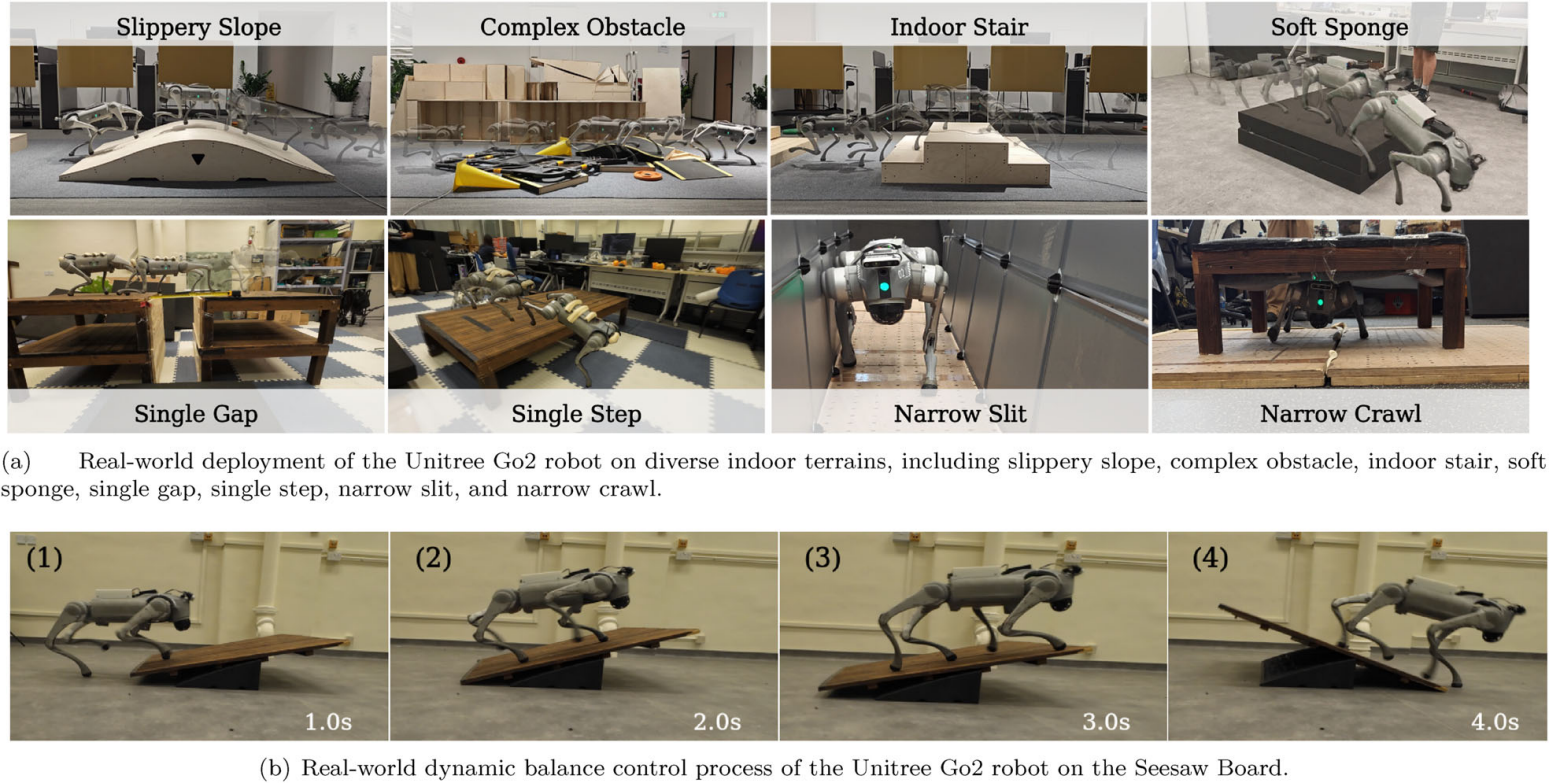
Robustness testing of the Unitree Go2 robot in diverse indoor terrains.

To establish the platform transferability and comprehensive generalization capability of our MGDP framework, we conduct a complementary series of real‐world validation tests using the Unitree Go2 robot across a diverse set of challenging indoor terrains, as visually summarized in Figure 10a. This rigorous test suite, comprising eight distinct structural and dynamic obstacles, is designed to collectively challenge specific locomotion requirements: the Slippery Slope tests frictional limits, while the Soft Sponge challenges compliance control on yielding ground; the Complex Obstacle requires stable gait maintenance and limb coordination within a cluttered layout; the Indoor Stair and Single Step assess precise foot placement and vertical clearance; the Single Gap and Narrow Crawl require precise coordination and collision avoidance; and the Narrow Slit evaluates posture control in constrained spaces.

In addition, to verify the robot's dynamic balance control capabilities, we conduct targeted experiments on a seesaw, as shown in Figure 10b. This figure records the adjustment sequence of the Unitree Go2 robot during the test, which includes (1) initial foot placement precisely on the horizontal seesaw; (2) countering slight tilt by adjusting joint stiffness and foot force; (3) coordinating front and rear leg movements to address significant seesaw tilt; and (4) completing traversal and transitioning to static ground.

Overall, these detailed indoor evaluations verify the policy's ability to maintain high gait stability and precise movement across diverse indoor structures, conclusively demonstrating MGDP's platform generality for deploying learned locomotion behaviors in structurally and perceptually demanding real‐world indoor environments.

### 7.9 Real‐World Transfer on Outdoor Terrains

**FIGURE 11:**
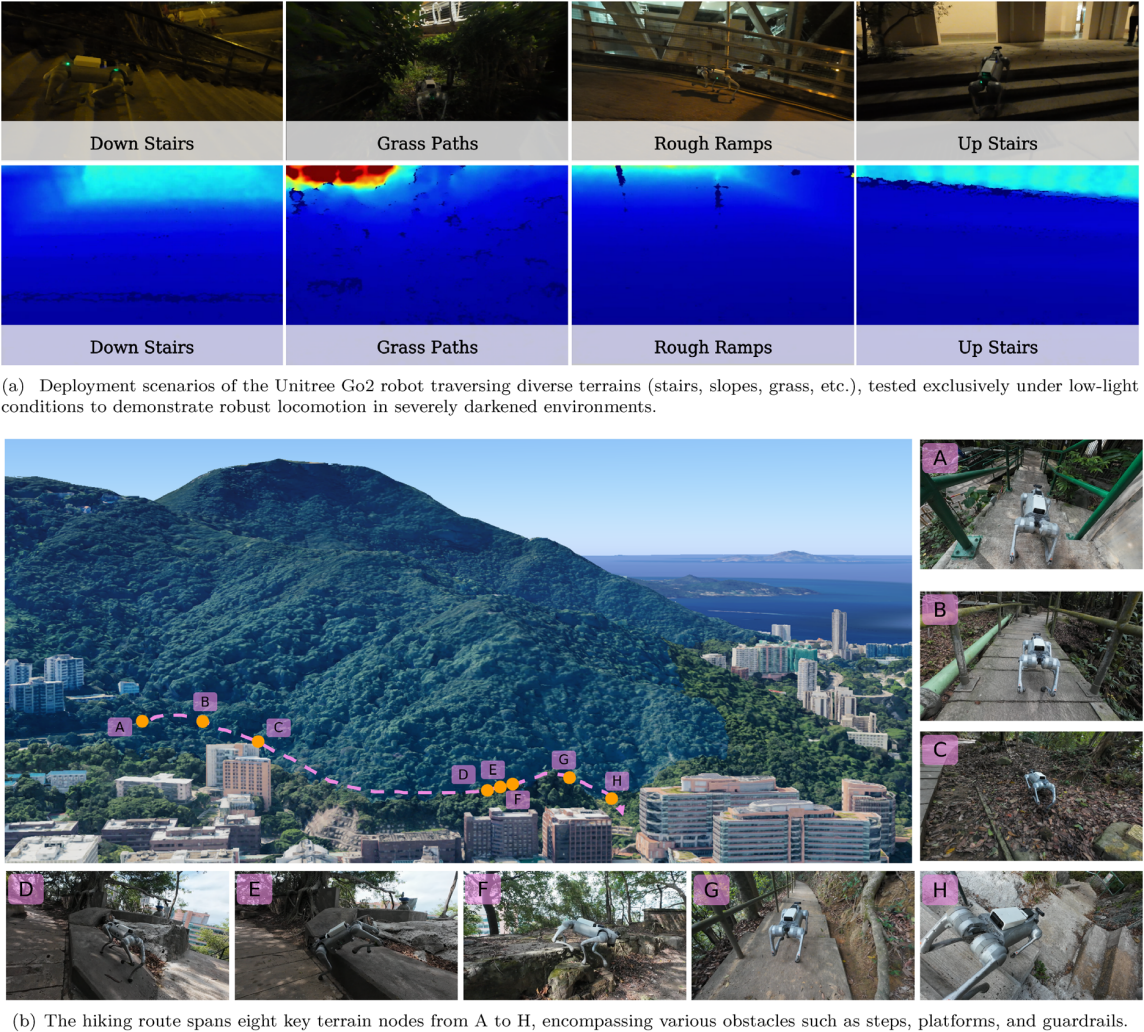
Robustness testing of the Unitree Go2 robot in diverse challenging terrains: (a) Outdoor experiments on the campus routes, (b) Robustness tests on the hiking route in a park.

To validate the real‐world transfer capability of our MGDP, we perform comprehensive and robust testing of the Unitree Go2 robot in diverse, challenging outdoor terrains, as summarized in Figure 11. In Figure 11a, we present deployment scenarios of the quadrupedal robot Go2 across diverse unstructured outdoor terrains (e.g., slopes, natural grass, and stairs). Crucially, these tests are conducted exclusively under low‐light conditions and artificial nighttime illumination. This rigorous setup is designed to systematically evaluate the performance of the MGDP depth perception model against the inherent challenges of real‐world sensor noise and poor light contrast in darkened environments. These experiments collectively evaluate the performance of MGDP in complex real‐world settings and conclusively demonstrate its high adaptability to different environmental and topographical conditions.

Figure 11b illustrates a long‐duration hiking route, spanning eight key terrain nodes from A to H. This route strategically encompasses various sequential obstacles such as steps, uneven platforms, and edge‐following along guardrails. Through these sustained tests, we assess not only the robot's ability to cross individual obstacles but its long‐term stability. This verification process confirms MGDP's capability to transfer learned strategies to unstructured outdoor environments for prolonged operation.

### 7.10 Statistical Analysis

(1) Pre‐processing of data: Simulation data (learning curves, success rates, loss curves) are recorded per episode or evaluation run without further pre‐processing. (2) Data presentation: Quantitative results are reported as mean ± standard deviation where applicable. (3) Sample size (n): Learning curves and reward comparisons use n=5 independent random seeds per method (4096 parallel environments per seed); the metrics in the ablation experiments are evaluated over multiple robot platforms and 10 terrain types across 10 difficulty levels; loss curves show the mean over the training batch. (4) Statistical methods: Comparisons are descriptive; no formal hypothesis testing (e.g., no P values or significance tests) is performed, in line with common practice in reinforcement learning and robotics. (5) Software: All analyses and plots are generated in Python, using PyTorch and Isaac Gym.

## 8 Conclusion

In this work, we proposed a novel DRL control framework, MGDP, fundamentally designed to address core bottlenecks in existing depth‐perception policies: high computational overhead, sensor noise sensitivity, and depth hardware coupling. We leveraged contrastive learning to extract low‐dimensional, generalized terrain features from multi‐modal inputs, achieving an effective decoupling of terrain perception from robot dynamics, which is further complemented by an explicit depth map denoising mechanism to enhance policy robustness. Furthermore, we utilized an optimized parallel computation architecture to significantly mitigate the high computational cost. Extensive experimental results strongly validated that the MGDP framework endowed the policy with superior cross‐terrain generalization capability, demonstrating a dominant advantage, particularly in complex environments. Most importantly, by utilizing the pre‐trained MGDP perception model, the policy can be quickly and efficiently fine‐tuned across quadruped robots of varying morphologies and hardware configurations. This work provides critical support for building unified and highly transferable locomotion controllers for legged robots. In the future, we intend to focus on two major directions. First, to bridge the simulation‐to‐reality gap and further enhance the generalization of our perception module, we will collect real‐world depth images captured during robot locomotion and utilize this empirical data to optimize our perception model. Second, we aim to continually refine our framework to achieve a truly unified and zero‐shot generalized controller. This ambitious goal involves developing a single policy capable of adaptively controlling legged robots of various gaits and morphologies, including even humanoid robots, enabling successful traversal across diverse terrains without requiring any further fine‐tuning.

## Conflicts of Interest

The authors declare no conflicts of interest.

## Data Availability

The data that support the findings of this study are available from the corresponding author upon reasonable request.
